# Systematic Review: Long-Read Sequencing in Algal Studies

**DOI:** 10.3390/ijms27052415

**Published:** 2026-03-05

**Authors:** Kakima Kastuganova, Alyamdar Askerov, Attila Szabó, Natasha S. Barteneva

**Affiliations:** 1 Department of Biology, School of Sciences and Humanities, Nazarbayev University, Astana 010000, Kazakhstan; kakima.kastuganova@alumni.nu.edu.kz (K.K.); alyamdar.askerov@nu.edu.kz (A.A.); 2 Department of Aquatic Sciences and Assessment, Swedish University of Agricultural Sciences, 75007 Uppsala, Sweden; attila.szabo@slu.se

**Keywords:** long-read sequencing, nanopore sequencing, metabarcoding, 16S/18S rRNA gene, pangenome, algal host–bacterial symbiosis, algal microbiome, ONT, PacBio

## Abstract

Long-read sequencing (LRS) has transformed life science research by introducing third-generation sequencing (TGS) platforms applicable across various research fields, including environmental sciences. In the past decade, LRS platforms have been utilized to extensively study algal systems by improving genomic approaches such as metabarcoding, chromosome-level genome and pangenome assemblies, as well as providing new insights into algae-associated microbiomes and host–symbiont interactions. This review aims to discuss recent advancements in LRS in algal research. To achieve this aim, a systematic review was conducted according to the PRISMA 2020 guidelines and across three electronic databases (Web of Science, Scopus, and Google Scholar), with additional citation searching for relevant studies in four key algal research areas: metabarcoding, genomics, pangenomics, and host–symbionts interactions. Following the inclusion and exclusion criteria, only 51 studies were selected for this review. Throughout the review, we summarize the challenges of short-read sequencing (SRS) and discuss how LRS platforms address these challenges in algal studies. Furthermore, we discuss the future of LRS and explore how artificial intelligence (AI) can advance research on algal biology and ecology.

## 1. Introduction

During the past decade, algae have been studied primarily using “short-read” sequencing Illumina-based platforms relying on platform-specific library preparation protocols. However, due to short-read length (i.e., app. 100 to 300 bp sequences), Illumina-based platforms currently face challenges in PCR bias, high levels of sequence biodiversity, reduced coverage of GC-rich regions, and difficulties in resolving highly repetitive regions, paralogous regions of the genomes, structural variations, index hopping, and large segmental duplications [1,2,3,4,5,6]. These challenges combined can now be addressed with the advent of long-read sequencing (LRS) and third-generation sequencing (TGS) tools.

**Figure 1:**
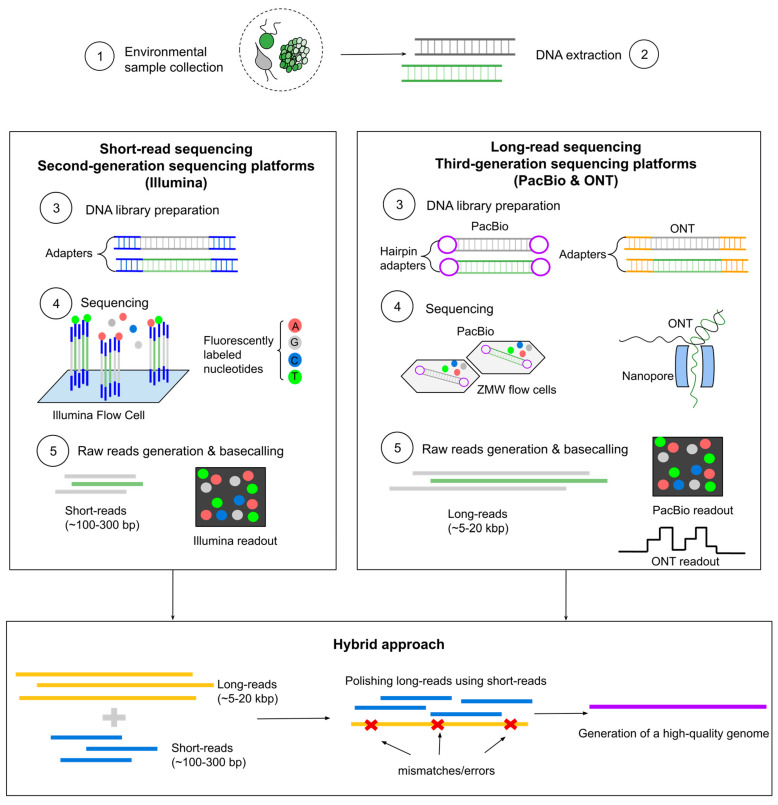
The comparison of second-(Illumina) and third-generation (PacBio and ONT) sequencing workflows: (1) Environmental sample collection; (2) DNA extraction and purification; (3) Library preparation (Illumina and ONT use adapter-ligated libraries, while PacBio uses hairpin-ligated Single Molecule Real-Time (SMRTbell) library); (4) Sequencing methods (Illumina uses sequencing by synthesis with bridge clusters and addition of fluorescently labeled nucleotides; PacBio uses single-molecule sequencing on a Zero Mode Waveguide (ZMW) flow cell; ONT uses nanopore channels for single-molecule sequencing); (5) Raw reads generation and basecalling: (Illumina generates short reads (~100–300 bp) based on fluorescence; PacBio and ONT generate long reads (~5–20 kbp), with PacBio using fluorescence and ONT measuring current changes as a readout). The hybrid approach combines both methods, producing short and long reads where short reads polish long reads from mismatches/errors in the DNA sequence for high-quality genome assembly.

TGS technology refers to single-molecule sequencing technology which can accurately sequence long strands of nucleic acid without an intermediary and without previous retro transcription or amplification [7]. Two popular LRS platforms, Oxford Nanopore Technology (ONT) and Pacific Biosciences (PacBio), provide median length of reads from 5 to 20 kbp and throughputs from 15 to 50 Gbp [8], covering full-length 16S-, 18S rRNA genes and ITS (internal transcribed spacer) region for prokaryotic and eukaryotic species [9,10] (Figure 1). The length of LRS depends on the specific technology and instrument platform used. The longer reads offer several advantages over short-read sequencing (SRS) methods, resulting in fewer sequencing fragments with wider coverage, accurately, assembling genomes and minimizing errors in genome assembling, particularly in highly repetitive regions [11]. Larger variations in the sequence are difficult to detect with short reads; second-generation sequencing (SGS) is well suited for identifying single-nucleotide variations (SNVs) and small insertions and deletions (indels), but not larger variations in the sequences [12,13].

The criticisms towards LRS were due to its early, relatively low accuracy, which has significantly improved with advancements in different aspects of nanopore sequencing, including nanopore sensors and associated chemicals, speed, and the ability to handle larger sets of reads [14]. The error rate in ONT is related to its capability to control the speed of DNA molecules passing through the nanopore, whereas the PacBio error rate is affected by random errors [15,16]. The integration of phi29 DNA polymerase and introduction of new flow cells reduced the error rate in ONT [17]. Furthermore, PacBio implemented circular consensus sequencing, and PacBio’s Revio iteration has reached an accuracy level of 99.9%, closely comparable with SRS [18,19]. The notable drawback of LRS when compared to SRS is its relatively higher sequencing cost, which continues to be more expensive despite all advancements in technologies. The relatively low cost and portability of sequencing module from ONT (MinION) [20] (though not consumables) made use of the technology possible by individual laboratories and not by core facilities. It was particularly important for environmental scientists who traditionally rely on smaller grants. There is an increased interest in applying LRS in studying algal communities. Research groups have reported using these platforms for algal taxonomic profiling [21,22], characterizing species of harmful algal blooms (HABs) [23,24,25,26], analyzing algal-associated microbiomes [27,28,29,30], assembling algal genomes at the chromosome scale [31], studying algal pangenomes [32,33,34], and investigating algal host–symbiont interactions [35,36,37].

Currently, however, broad, comprehensive reviews on the use of SGS in algal systematics are available [38,39,40] or studies on certain aspects of LRS, such as metagenomics [7] or algal microbiomes [28]. To systematize current advances in LRS in studies of algal ecosystems, we focused on the following four critical areas of algal research: algal metabarcoding, algal genomics, pangenomics, and algal host–bacterial symbiont studies. We also investigated the current performance of SGS compared to traditional visual methods, such as microscopy, and discussed challenges and future directions for integrating LRS data into multi-omics studies.

## 2. Materials and Methods

This systematic review was conducted in accordance with the Preferred Reporting Items for Systematic Reviews and Meta-Analyses (PRISMA) guidelines [41]. The focus of the review was long-read sequencing in algal systems, not in clinical settings; thus, it was not registered.

### 2.1. Search Strategy

For this review, we focused on studies that examined the use of common LRS platforms (ONT, PacBio) in the following areas: algal metabarcoding, algal genomics, algal pangenome, and algae-host bacterial symbionts. To identify relevant studies, we conducted an initial search from 8 to 9 September 2025, across several electronic databases: Web of Science (WoS), Scopus, and Google Scholar. The following search terms were used to cover all relevant studies: algae (“algae”, “algal”), metabarcoding (“18S”, “16S”), genomics (“long read genome sequencing”, “whole genome”, “whole genome sequencing”, “genome”), pangenome (“pangenome”, “pan-genome”), host–symbiont (“symbiont”), and long-read sequencing (“nanopore sequencing”, “long read nanopore sequencing”, “PacBio”, “ONT”, “long read sequencing”, “next generation sequencing”, “long read”). The general search query we employed across all databases was algae AND area (metabarcoding OR genomics OR pangenome OR host-symbiont) AND long-read sequencing. The time limit for publications was set to 2015–2025. To ensure that we included articles that may have been missed in the initial search, we conducted an additional manual search. Only a select few studies comparing SGS with traditional methods were identified through citation searching, and these studies were not included in a systematic review because they were outside the scope of the research topic.

### 2.2. Inclusion and Exclusion Criteria

Following the removal of duplicates, articles were included for further screening if they met the following inclusion criteria: (1) written in English; (2) original research articles; (3) focused on algae-based system; (4) employed long-read sequencing platforms to study algal metabarcoding, genomics, pangenome, or algae host–bacterial symbionts; and (5) provided sufficient methodology reporting. Articles were excluded if they were not written in English, were not published as research articles (e.g., reviews, manuscripts, book chapters, conference papers, etc.), or were not relevant to the research topic and algae-based study system.

### 2.3. Data Extraction

We collected the following data for each publication: the first author’s name, the year of study, the algae system, DNA extraction method, the library preparation protocol, the sequencing platforms used, the read length (paired-end or single-end), and the sequencing depth/coverage per sample. Additionally, we included data on genome assembly and polishing tools for genomics studies and pangenome pipeline tools for pangenome studies.

### 2.4. Study Selection

**Figure 2:**
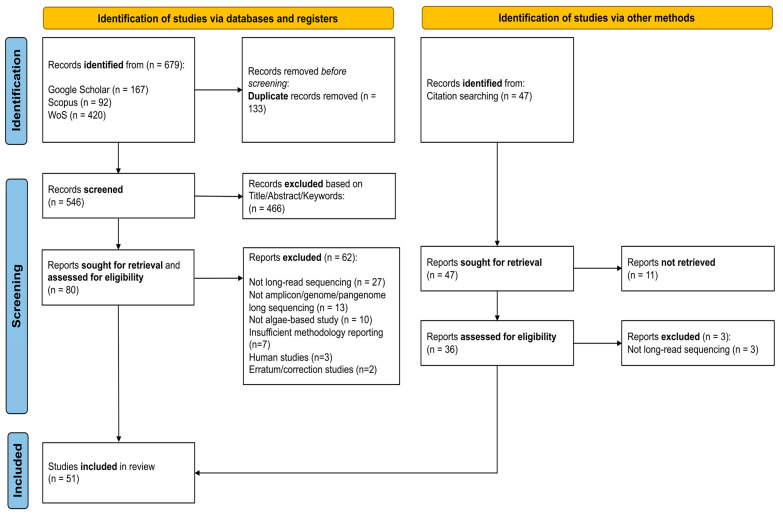
PRISMA (Preferred Reporting Items for Systematic Reviews and Meta-Analyses) flowchart summarizing the steps of article identification, selection, screening and inclusion stages. This systematic review adapted the PRISMA guidelines. This review, however, did not include clinical data and, therefore, was not registered.

Our systematic review focused on examining long-read sequencing in algal metabarcoding, genomics, pangenomics, and host–symbiont studies. The comprehensive search strategy allowed us to retrieve 679 articles from three databases (WoS, Scopus, and Google Scholar), and an additional manual search resulted in 47 studies (Figure 2). After removing duplicates, 593 articles were screened based on title, abstract, and keywords. At this stage, the articles were removed based on language, article type, and relevance to the four key research areas. This screening procedure resulted in the removal of 477 articles. During the inclusion step, the remaining 116 articles were assessed based on the inclusion criteria outlined in the Materials and Methods Section 2, resulting in the removal of 65 articles for different reasons highlighted in Figure 2. After this step-by-step selection process, a total of 51 articles were included for systematic review analysis. Of these studies, 25 articles were algal metabarcoding studies, 14 articles were algal genomics studies, 6 articles were algal pangenome studies, and 6 articles were algal host–symbiont studies.

## 3. Results and Discussion

### 3.1. Long-Read Metabarcoding

Algal metagenomics (untargeted sequencing of DNA from all organisms associated with a given algal sample) [42] and 16S rRNA and 18S rRNA gene amplicon sequencing have become instrumental methods in determining algal diversity, monitoring HABs, and characterizing algal microbiomes [28,30,43,44]. As proof of concept, LRS’s potential to detect toxic algae was observed in mock community studies, where the MinION (ONT) platform demonstrated capabilities to determine dinoflagellate species belonging to *Alexandrium* genus [44]. There is a wide collection of studies available that confirmed LRS capacity for detection and monitoring potentially toxic cyanobacteria (e.g., *Microcystis*) [21]. Baharudin and co-authors [26] have utilized LRS for monitoring toxic algae belonging to dinophytes in shrimp aquaculture ponds, highlighting its utility as a warning system in aquaculture industries that are often susceptible to HAB outbreaks. Moreover, several studies report the use of LRS in assessing microbial communities associated with causative agents such as *Microcystis* [30] and toxic dinoflagellates (*Alexandrium tamarense* and *Cochlodinium polykrikoides*) [27], which underscores the potential of LRS in evaluating complex HAB dynamics.

Currently, SRS is commonly used in algal metabarcoding, targeting specific hypervariable regions in two marker genes, the 16S and 18S rRNA genes, for the determination of cyanobacteria and eukaryotic algae [5,45]. The 16S rRNA is a gold standard for bacterial community profiling due to ubiquity, the low frequency of horizontal gene transfer (HGT), taxonomic information from hypervariable regions (V1–V9), and the presence of universally conserved regions that serve as primer-binding sites for PCR. The Tara Oceans expeditions produced a very large metabarcoding dataset capturing the highest diversity and large number of sequences from undescribed species among picoplankton groups [46]. The utility of metabarcoding and metagenomics lies in their potential to provide quantitative measures of algal biodiversity, albeit without characterizing newly discovered species beyond the sequences obtained [39]; however, it is increased by a hybrid sequencing approach (SRS and LRS together) [47].

Metagenomics answers research questions in two different ways: (1) shotgun metagenomics and (2) amplicon sequencing. In amplicon sequencing, the application of the method, called “meta-profiling”, utilizes a single standardized genetic marker, amplified to identify multiple species simultaneously from complex samples [48]. The whole genome shotgun sequencing approach utilizes total DNA, which is randomly fragmented during the library preparation step, fragments of which are sequenced in parallel, and later are assembled into contiguous sequences based on overlapping regions. Moreover, such approaches in SRS platforms may target short-amplicon sequences not covering the whole gene and usually extend only to several hundred bps [49,50,51,52]. Thus, for the 18S ribosomal rRNA gene, regions commonly used to characterize the diversity of eukaryotic communities by metabarcoding are the V4 and V9 gene regions.

**Figure 3:**
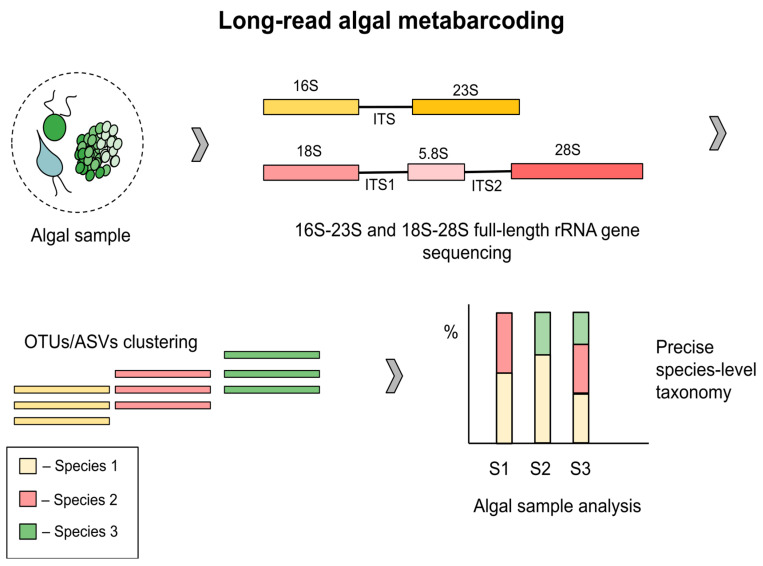
Overview of long-read sequencing in algal metabarcoding. The step-by-step procedure: (1) algal sample collection; (2) full-length rRNA gene sequencing (16S-ITS-23S and 18S-ITS-28S); (3) OTUs/ASVs clustering; (4) taxonomic profiling at the species level. (ITS—internal transcribed spacer, OTUs—operational taxonomic units, ASVs—amplicon sequence variants, S—sample).

Additionally, short-reads have difficulty reliably resolving taxonomic assignments at the species level, with most short-read studies showing a consistent decrease in taxonomic resolution from genus to species levels [49,50,51,53,54]. The growing preference for full-length 16S rRNA and 18S rRNA gene sequencing using long-read platforms reflects a trend towards species- and potentially strain-level resolution in microbial and microalgal research [55,56,57]. With the advent of LRS platforms, it has become possible to address the issues. Currently, LRS platforms are capable of targeting full-length 16S and 18S rRNA, with the possibility to sequence the entire rRNA genes 16S-ITS-23S [58] and 18S-ITS1-5.8S-ITS2-28S (Figure 3, Table A1). However, species-level identification is not always achievable, and early versions of ONT platforms were hampered by reads of low accuracy.

While partial 16S rRNA gene regions are generally sufficient for identifying of bacterial heterotrophs, sequencing the full-length 16S rRNA gene provides improved taxonomic resolution [59]. However, the reliable identification of oxygenic phototrophs may require additional 18S rRNA nuclear (nuITS1, nuITS2), plastid (*rbc*L, *tuf*A, and Cp23S), and mitochondrial genes (cytochrome *c* oxidase subunit (COI)) [60,61,62]. However, DNA barcoding of microalgae remains limited by the lack of universally applicable markers that could distinguish closely related species. Using multiple genes for barcoding appears to be more effective [60,61,62]. Different groups of microalgae are often targeted with different barcode markers for SRS. Thus, for diatoms the best functioning barcode markers were reported as follows: (i) the 3′ end of the large subunit of the *rbc*L (*rbc*L-3 P), (ii) a 540 bp fragment situated 417 bp downstream of the start codon of the *rbc*L (540 bp *rbc*L), (iii) the 5′ end of the mitochondrial cytochrome c oxidase I gene (COI-5 P), (iv) a partial sequence of the large ribosomal subunit (D1–D3 LSU, usually either D1–D2 or D2–D3), and (v) the V4 sub-region of the small ribosomal subunit (V4 SSU) [63]. Recent advances in LRS accuracy have prompted the use of parallel sequencing for mitochondrial DNA (mtDNA) and other amplicons in biodiversity research. Thus, Karin and co-authors [64] and Vossen & Buermans [65] employed a methodology involving two asymmetrically indexed and overlapping fragments of mtDNA.

Notably, some of these studies compared the performance of short- and long-read sequencing in resolving taxonomic levels, especially at the genus and species level [26,27,66,67,68,69,70]. These comparisons have shown that LRS provides better taxonomic resolution at the species level [26,27,66]. Thus, Latz and co-authors [66] compared PacBio vs. Illumina platforms (MiSeq and NovaSeq) and reported that long-read amplicons and ITS1 alone provided higher taxonomic resolution than V4 region primers.

We examined SRS in comparison with traditional methods that rely on morphology and microscopy. Specifically, the concordance between these two methods was determined by the percentage overlap of taxonomic assignments at the genus and species levels. Importantly, to the best of our knowledge, the current literature is rich in comparative studies focusing on SRS, whereas such comparisons remain limited for LRS. Nevertheless, this analysis is essential in demonstrating the current performance of short-read-based tools compared to traditional morphological approaches.

**Figure 4:**
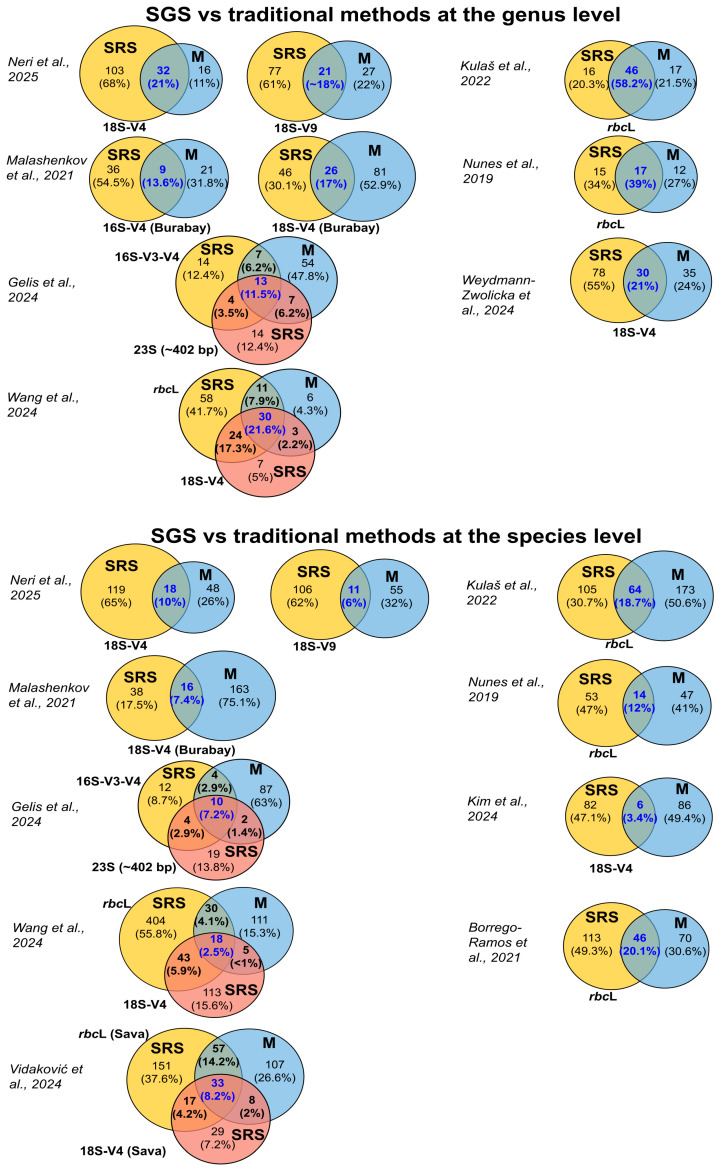
Overlap % of the identification of algal taxa by SGS versus traditional methods at the genus and species levels (SRS—SRS-based methods, M—microscopy- or morphology-based methods). For studies with multiple comparative analyses, a single representative analysis was included [49,50,51,52,53,54,71,72,73,74].

Our analysis revealed that at the genus level, short-read-based sequencing demonstrates better concordance with traditional methods, averaging between 11.5% and 58.2%, than at the species level, between 2.5% and 20.1% (Figure 4) [49,50,51,53,71,72,73,74]. One possible explanation for this high variation is that different studies targeted different marker genes (16S V3-V4, 18S V4/V9, 23S, *rbc*L).

At the species level, the overlap percentage across all studies decreased significantly, especially in those studies that conducted both genus- and species-level comparisons (Figure 4).

LRS and use of full-length 16S rRNA gene provided a higher proportion of reads that were further assigned to the species level [66,75,76]. However, the LRS’s potential to determine finer taxonomic levels is currently hindered by gaps in reference databases and in bioinformatics pipelines [68,69].

### 3.2. Long-Read Algal Genomics

With the advent of next-generation sequencing (NGS) platforms, whole-genome sequencing (WGS) has become the standard procedure in genomics [77] and environmental [78,79,80,81,82,83,84,85] research. Short-sequencing platforms made WGS widely acceptable, enabling the sequencing and assembly of many novel genomes and the generation of accessible genome data [86,87,88]. Billions of short reads spanning the length up to 300 bp can reliably provide information on single-nucleotide variants, copy-number variants, and insertions or deletions (indels) [89,90,91]. However, SRS platforms currently face challenges in resolving highly repetitive regions, genome structural variations, segmental duplications, and GC-rich regions [2,3,4,92].

**Figure 5:**
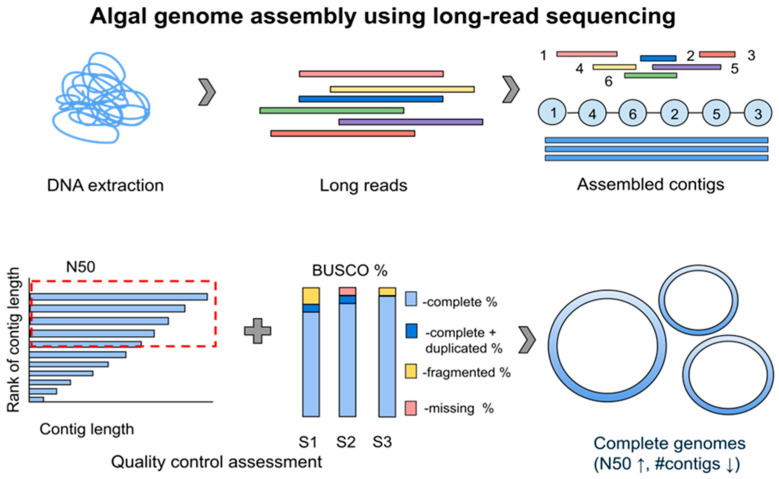
Overview of long-read sequencing in algal genomics. The step-by-step assembly is illustrated in the following steps: (1) DNA extraction from an algal sample; (2) generation of long-reads using ONT and/or PacBio; (3) assembly of contigs from long-reads (as illustrated 1-6 long reads are assembled according to their overlapping regions to produce contigs); (4) quality control assessment based on N50 and BUSCO % (C); (5) assembly of complete genomes with a high N50 metric and a low number of contigs. (N50—contig length representing 50% of the total (contiguity), BUSCO—Benchmarking Universal Single-Copy Orthologs (completeness), S—sample).

In recent decades, LRS platforms have made great advancements in resolving these issues. LRS can generate reads ranging from 10 kilobases to several megabases, enabling the production of continuous sequences directly from native DNA [2]. The major advantage of ONT and PacBio technologies in constructing complete genomes is the ability to reduce the number of contigs while increasing the N50 metric (Figure 5, Table S1).

This capability of LRS is evident in several studies that report highly contiguous and complete genomes for lichenized algae, HAB-forming, and psychrophilic organisms, as well as algicidal and intertidal algal species [31,93,94,95,96,97,98]. Numerous studies have successfully generated near-complete genomes at the chromosome scale and even at the telomere-to-telomere (T2T) scale [81,94,97,99], as evidenced by their high Benchmarking Universal Single-Copy Orthologs (BUSCO) scores, which provide evolutionarily sound quantitation of completeness and redundancy in terms of expected gene content [100]. Although LRS, in comparison to SRS, generates highly contiguous genomes, its per-read sequencing accuracy and the quantification of LRS data remain challenging.

Constructing a complete algal genome allows us to understand evolutionary dynamics and investigate the putative genes behind environmental adaptations. Several studies have employed WGS to examine the evolutionary changes in genome structure and organization. These include revealing whole-genome duplication events in *Diplosphaera chodatii* Bialosuknia and retrotransposon proliferation in *Chlamydomonas* sp. ICE-L [96,101,102]. Some studies further extended the WGS to examine HGT events using a three-step approach: (1) identifying candidate HGT genes by querying databases for homologous sequences using DIAMOND or BLAST; (2) evaluating candidate genes with Alien Index analysis; (3) constructing a maximum likelihood tree. Following this approach, Jian and co-authors [23] identified HGT events in obligate algal heterotrophs. Notably, this study used the HGTphyloDetect tool to identify HGT events. This tool integrates the three aforementioned steps into a single program, enabling a comprehensive HGT analysis [101].

Furthermore, Zhang and co-authors [102] investigated HGT-acquired proteins from prokaryotic donors as potential drivers for the emergence of a psychrophilic lifestyle in *Chlamydomonas* sp. ICE-L. Additionally, Chen and co-authors [95], as well as Wang and co-authors [93], examined various environmental adaptations obtained through putative bacterial HGT genes [93,95]. Thus, the complete and contiguous genomes generated through LRS significantly contribute to our understanding of the evolution and adaptation of algal lineages.

### 3.3. Pangenome Long-Read Sequencing

Delineating species boundaries has been a long-standing challenge in algal studies [103,104]. Methods of species delimitation in algae relying on morphology or sexual compatibility have gradually been replaced by molecular sequence data [105]. It is particularly important for small and morphologically indistinguishable organisms [106,107], or algae with high morphological variability and plasticity [108,109,110,111], such as colonial *Microcystis* Lemmermann. Several statistical approaches have been developed, including (1) distance-based approaches to determine barcode gaps, defined as the difference between intra- and interspecific genetic divergence [112,113], and (2) tree-based methods [114,115]; both approaches are typically applied to single-locus datasets. On the other side are multi-locus delimitation methods, based on the (3) multispecies coalescent framework that include Bayes Factor delimitation [116] and SpedeSTEM [117].

**Figure 6:**
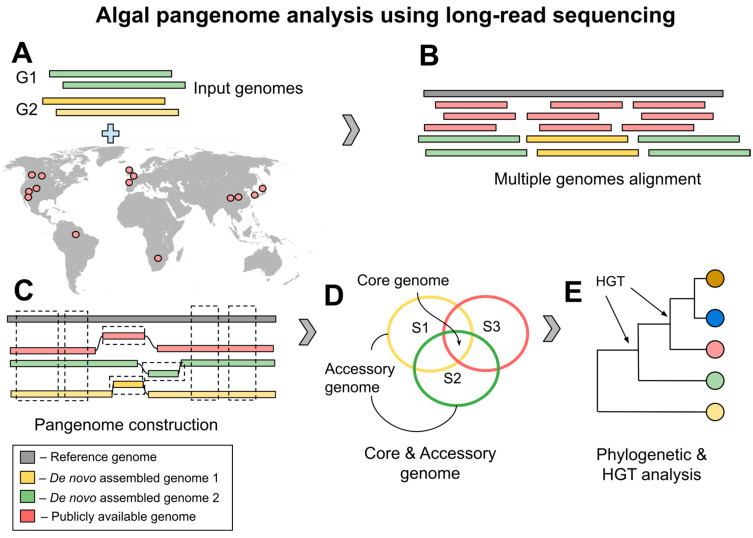
Overview of long-read sequencing in algal pangenome studies. (**A**) Collection of genome data for pangenome analysis based on *de novo* assembly (G1–G2) and publicly available data (illustrated in the map); (**B**) multiple alignment of all included genomes against known reference genome data; (**C**) pangenome construction, with the dotted areas illustrating the shared/core genome; (**D**) Venn diagram of core and accessory genome data; (**E**) phylogenic analysis for species delimitation and HGT analysis to determine evolutionary and environmental adaptations (S—species, HGT—horizontal gene transfer).

Pangenome studies allow reclassification of species, thereby changing and improving traditional taxonomic criteria [118]. It also allows cataloging multiple genomes from a species and identifies shared genes across lineages that are responsible for essential metabolic processes, as well as accessory genes in specific lineages that contribute to adaptive traits [119]. LRS platforms have become instrumental in establishing pangenomes, delineating species, identifying core and accessory genomes, and tracing HGT events across a phylogenetic tree (Figure 6). One major conclusion from pangenome research is that no single strain can ever be considered the typical reference since not all genes will be present in all strains, and perhaps not in the same order [120].

In pangenome studies, an efficient sampling strategy is crucial to maximizing biogeographic and genomic diversity [121]. To investigate this diversity, some studies utilized available complete and draft algal genomes from NCBI databases [32,122,123], while others sampled algae from their local environments and generated genome assemblies using a hybrid short- and long-read sequencing approach [33,34,124,125]. Irrespective of the strategy employed, the geographic sources of these algal samples are predominantly concentrated in the USA, Canada, and Europe, followed by East Asian countries (Table S2). This pattern aligns with the sampling bias observed in many pangenome studies, highlighting the challenges associated with the overrepresentation of some regions [126]. However, making affordable LRS platforms, such as MinION (ONT), accessible to underrepresented regions could help bridge the sampling gap, providing missing data for ongoing pangenome projects.

Several studies have utilized LRS to construct pangenomes of HAB-forming species, including *Microcystis*, *Prymnesium parvum* N.Carter, and *Aureococcus anophagefferens* Hargraves & Sieburth [32,33,34,123,125]. Studies highlighted the remarkable plasticity of the *Microcystis* pangenome, reporting that 92.5% of the genes are dispensable and revealed the presence of 16 putative genospecies [32,123]. Similarly, the haptophyte HAB-forming *P. parvum* pangenome has a significant dispensable genome and includes at least three cryptic morphospecies [33]. On the other hand, haptophyte *A. anophagefferens* has a highly conserved core genome [34,125].

Many reported accessory genes in the dispensable genome may have originated from HGT events [33,34,122,123,127]. Using a three-step approach, these studies identified HGT events from prokaryotic, viral, and eukaryotic donors responsible for toxin metabolism in *Microcystis* and environmental adaptations in various algae, including shifts in lipid metabolism, halogenated hydrocarbons, carbon acquisition, and stress response. However, there is ongoing debate about whether some of these genes can truly be classified as HGT-derived. Challenges include gene erosion, bacterial contamination, and differential loss of genes during eukaryotic HGT [127]. The use of LRS can facilitate an acquisition of long fragment information and sequence regions that are problematic with short-read sequencing approaches. Overall, eukaryotic HGT events represent a minor percentage in whole pangenome analyses (e.g., HGT-derived genes in Cyanidiales: 1% [127]; in cryptophytes, rhizarians, alveolates, stramenopiles, and haptophytes (CRASH taxa group): 0.16–1.44% [122]). Despite their rarity, these HGT events may provide insight into evolutionary adaptation and contribute to the genetic and functional diversity observed in algal species.

### 3.4. Algal Host–Bacterial Symbiont Long-Read Sequencing

Bacteria for a long time have been considered as contaminants in microalgal cultures, with efforts directed toward obtaining axenic algal monocultures. However, the recognition of mutualistic microalgae–bacteria interactions has opened opportunities to use bacterial exosymbionts as growth-promoting partners in microalgal cultivation [128,129]. These species-specific phytoplankton–bacteria interactions should often be considered as symbiotic [130,131].

The successful separation of the host genome from prokaryotic contamination and associated symbionts presents a significant challenge in genomic studies. Several strategies have been developed to address this issue in algae [35,93,132,133,134] and computational tools developed to distinguish hosts from associated microbes and symbionts [134,135]. While combining these strategies with SRS has led to notable improvements in host–symbiont studies, major challenges remain in resolving structural variations (inter- and intra-genomic repeats, segmental duplications) of host–symbiont genomes [128]. LRS offers potential solutions to these challenges by facilitating the disentangling of the genomes of the algal host from the symbionts and associated microbial species.

**Figure 7:**
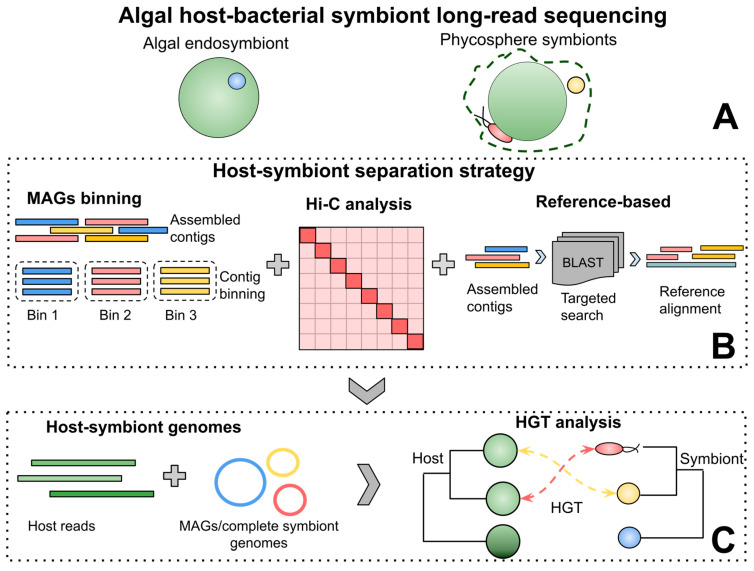
Overview of long-read sequencing in algal host–bacterial symbiont studies. (**A**) Two common symbionts reported in algae—endosymbionts and symbionts—inhabiting the diffuse area surrounding phytoplankton (single cell or colony) known as the phycosphere. (**B**) Host–symbiont separation strategies: (1) MAGs binning: Clustering assembled contigs into representative bins based on nucleotide composition and coverage. (2) Hi-C analysis: Visual segregation of genome regions to reveal the genome’s 3D structure. (3) Reference-based approach: Assembled contigs undergo a targeted search and are evaluated against the reference genome. (**C**) Generation of symbiont-free host genomes and complete MAGs/symbiont genomes, and HGT analysis to characterize evolutionary dynamics and environmental adaptations. (MAGs—metagenome-assembled genomes, HGT—horizontal gene transfer).

Algae can host bacterial endosymbionts or a diverse group of exosymbionts inhabiting the phycosphere, a diffuse area immediately surrounding an algal cell (Figure 7). The symbiotic partners may provide various services to their algal hosts, such as nutrient exchange, and help algal hosts survive in environments that experience light- and heat stress [37,136,137,138,139]. The genomic basis of such symbiotic partnerships can be effectively studied using LRS and refined host–symbiont separation strategies, including metagenome-assembled genomes (MAGs) binning, Hi-C analysis, and a reference-based approach (Figure 7). Several research groups have implemented a combination of short and long reads to generate complete symbiont genomes via MAGs binning using tools such as MyCC, MaxBin2, and MetaBAT2 [140,141,142].

In some studies, researchers combined PacBio high-fidelity reads and ONT reads with Hi-C analysis to visually segregate the host genome from symbionts [137,139]. For a more targeted approach, researchers preferred a reference-based strategy for isolating the symbiont’s genome, utilizing a BLAST search of long contigs against a reference genome [136,137,143]. Notably, the study by Wang and co-authors [137] employed the newly developed reference-free tool Symbiont Screener for generating high-quality host reads free from symbionts and associated microbial partners using a trio-based screening model [35]. These approaches demonstrate the effectiveness of LRS in addressing challenges in host–symbiont studies, including separating host and symbiont genomes and managing high levels of contamination from symbionts and other prokaryotes.

LRS tools are also invaluable for uncovering HGT events and their contribution to host adaptation. Wang and co-authors [137] identified 286 HGTs in the intertidal alga *Pyropia haitanensis*, with 50% of these transfers originating from symbiotic bacterial partners in the phycosphere, specifically from the *Pseudomonas*, *Actinobacteria*, and *Bacteroidetes* taxa. Among them, *Saccharothrix* sp. was isolated and shown to be associated with major environmental adaptation to heat stress in *P. haitanensis.* Likewise, Xu and co-authors [139] found 100 high-confidence HGTs distributed across 13 chromosomes of the intertidal algae *Bryopsis corticulans* Setchell. Although the exact taxonomic classification of the bacterial symbiont was not reported, this bacterium was shown to have conferred a cryptochrome gene to *B. corticulans*, thereby providing environmental adaptation to light-fluctuating environments. Thus, the capacity of LRS tools to clarify symbiotic partnerships can be utilized to investigate HGT events between hosts and their associated symbionts, helping to elucidate the environmental adaptations of algal species. LRS platforms demonstrate a wide range of applications in algal systematics. The advent of LRS on the ONT and PacBio platforms has resolved many challenges associated with SGS methods in areas like algal metabarcoding, genomics, pangenomics, and host–symbiont studies. Moreover, these new platforms have created opportunities for transcriptomic analyses, enabling the sequencing of full-length RNA molecules.

Advances in SRS technology provided a powerful foundation for metagenomics and the generation of high-quality genomes. However, genome assembly from short reads ranging between 100 and 300 bp in size due to the presence of repeated sequences [144] may result in fragmented and incomplete genome assemblies [92,145]. Since adopting long-read sequencing ONT and PacBio platforms, the field of genomics has undergone a revolutionary transformation [7,146]. These technologies represent a third generation in sequencing, addressing many challenges in SGS, including PCR amplification bias, difficulties resolving repeat regions, structural variations, GC-rich regions, and achieving finer levels of taxonomic resolution [28]. A key limitation of LRS is the availability of high molecular weight DNA, ideally, with large fragment sizes.

Following the introduction of platforms by ONT and PacBio, the LRS market has grown significantly, now offering a diverse array of long-sequencing instruments. Presently, there are several approaches that utilize nanopore chemistry [147]. Notable alternatives include the following: (1) QitanTech (China) technology has recently developed QNome-3841 nanopore system [148,149,150]; (2) AxBio (China, USA) technology has launched in 2023 AxiLona AXP100 instrument, which allows full-length 16S rRNA gene sequencing, providing higher-resolution bacterial community analysis [151,152]; (3) Bionano (USA) technology uses optical image mapping based on fluorescently labeled polynucleotides passing through a nanopore [153]; (4) Quantapore (USA) have been around since 2009 and developed an optical Nanopore DNA sequencing platform [147]; (5) Cambridge Nucleomics (UK) is another company which uses nanopore chemistry for direct quantification of tandem repeats in native RNA [154]. In the future, these nanopore-based systems could diversify existing LRS tools, thus offering more opportunities for long-read sequencing tools for algal research and beyond.

The widespread application of artificial intelligence (AI) methods, such as machine learning (ML), is rapidly transforming the computational aspects of LRS, including basecalling [155,156,157], methylation detection [158,159,160], and predictive analytics [161,162]. In the field of metagenomics, AI is advancing the detection of microbial signatures from complex microbiomes, thereby facilitating environmental monitoring and the characterization of key bioindicator species [163,164]. Currently, there are studies reporting on the use of AI in algal systematics. Notably, some research describes the use of ML approaches in identifying key algal taxa [165]. Additionally, deep learning-based approaches, such as Tiara, are being utilized to differentiate between host genomes and symbionts [139,166]. Overall, integrating AI into LRS shows promise for improving taxonomic classification, generating high-quality assemblies, and facilitating comprehensive environmental monitoring.

Some limitations of LRS should be considered when evaluating these review findings. First, the review was focused on ONT and PacBio long-read sequencing technologies, leaving out new platforms developed in China (QitanTech, AxBio, Geneus Gseq-500), the USA (Bionano, Nabsys, Quantapore), and the UK (Cambridge Nucleomics), among others [147,167,168]. Moreover, several studies have already been published that use QNome-3841 (QitanTech) nanopore sequencing platform in algal systematics [169]. Secondly, there is a significant challenge to develop bioinformatic methodologies, algorithms, and pipelines for these emerging technologies, as well as new statistical methods for long-read data.

Last but not least, the quality and completeness of available databases limit the utility of LRS. This issue is particularly evident in algal metabarcoding projects, where misidentified algal species can become prevalent depending on the workflow and the quality of the database used [68,170,171]. Furthermore, existing algal genome resources are heavily biased toward a few selected algal lineages, with several algal groups either underrepresented or lacking reference genomes altogether [172,173]. These challenges underscore the need for well-curated algal databases to enhance the utility of long-read sequencing-based approaches.

## 4. Conclusions

Long-read sequencing offers a scalable, rapid, and effective approach, providing numerous opportunities for algal metabarcoding, genomics, pangenomics, and host–symbiont studies. By utilizing long-read sequencing in these critical areas of algal research, we can accurately profile key algal taxa at the species level, characterize the algal microbiome, delimit species boundaries, and investigate the complex interactions between hosts and their symbionts. Coupling the advantages of long-read sequencing with bioinformatics pipelines enables the exploration of HGT, adaptations to environmental changes, and the evolutionary dynamics of algal lineages. In the future, emerging tools and advances in AI are expected to significantly advance the capabilities of long-read sequencing across various research domains. The valuable insights gained from algal studies can also be applied to other areas of environmental and conservation research.

## Data Availability

No new experimental data were created or analyzed in this study. Raw data sharing is not applicable to this article.

## Supplementary Materials

The following supporting information can be downloaded at https://www.mdpi.com/article/10.3390/ijms27052415/s1. Reference [174] is cited in the Supplementary Materials.

## Abbreviations

The following abbreviations are used in this manuscript:

LRS	long-read sequencing
SRS	short-read sequencing
TGS	third-generation sequencing
SGS	second-generation sequencing
NGS	next-generation sequencing
WGS	whole-genome sequencing
ONT	Oxford Nanopore Technology
PacBio	Pacific Biosciences
HGT	horizontal gene transfer
SNV	single-nucleotide variation
HAB	harmful algal bloom
BUSCO	Benchmarking Universal Single-Copy Orthologs
T2T	telomere-to-telomere
MAG	metagenome-assembled genome
AI	artificial intelligence
ML	machine learning
ITS	internal transcribed spacer
*coi*	cytochrome c oxidase subunit 1
CTAB	cetyltrimethylammonium bromide
PE	paired-end
SE	single-end
mtDNA	mitochondrial DNA
eDNA	environmental DNA
N/A	not available for this study

## Appendix A

**Table A1:** Summary of long-read algal metabarcoding studies and associated wet-lab techniques.

Study	Sequencing Strategy (16S or 18S)	Reported Algae	DNA Extraction Method/Kit	Library Preparation (Long-Read vs. Short-Read, Primers)	Sequencing Platforms	Read Length/Paired-End or Single-End
Shin et al., 2018 [27]	16S rRNA gene V3-V4 region, 16S full-length rRNA gene	Algal microbiomes: *Alexandrium tamarense*, *Cochlodinium polykrikoides*	QIAamp DNA microbiome kit (Qiagen) #	Short-read: 16S rRNA gene V3-V4 region * Long-read: 16S full-length rRNA gene, S-D-bact-0008-c-S20/S-D-bact-1391-a-A-17 primers	Illumina MiSeq, MinION (ONT)	MiSeq: 2 × 250 bp PE MinION: 1242 bp SE
Zhu et al., 2018 [175]	18S full-length rRNA gene	Ulvophyceae, Trebouxiophyceae, Chlorophyceae, Dinophyceae, Eustigmatophyceae	Higher plant DNA kit (Omega) #	Long-read: 18S full-length rRNA gene, NS1F/NS8R and NS1F/1650R primers	PacBio Sequel	1650–1850 bp SE
Curren et al., 2019 [43]	16S full-length rRNA gene	40 genera, 46 species	Phenol:chloroform:IAA method	Long-read: 16S full-length rRNA gene *	MinION R9.4 (ONT)	1502 bp SE
Hatfield et al., 2020 [44]	18S-ITS-28S rRNA gene	*Alexandrium* genus	Power Biofilm DNA isolation Kit (Qiagen, Hilden, Germany)	Long-read: 18S-ITS1-5.8S-ITS2-28S rRNA gene, 18ScomF1/D2C primers	MinION R9.4 (ONT)	2928 bp SE
Schafran et al., 2020 [171]	16S full-length rRNA gene, 23S full-length rRNA gene	*Anabaena*/*Dolichospermum* spp.	E.Z.N.A. Plant DNA kit (Omega Bio-tek) #	Long-read: 16S full-length rRNA gene, 23S full-length rRNA gene, N/A	MinION R9 (ONT)	~1185 bp SE (16S), 2469 bp SE (23S)
Mirasbekov et al., 2021 [176]	16S full-length rRNA gene	Phylum level	PowerWater DNA Isolation Kit (Qiagen, USA)	Long-read: 16S full-length rRNA gene *	MinION R9.4 (ONT)	N/A
van der Loos et al., 2021 [28]	16S full-length rRNA gene	Algal microbiomes: *Ulva australis*, *Ulva lacinulata*	DNA PowerSoil kit (Qiagen) #	Long-read: 16S full-length rRNA gene, 27F/1492R primers	MinION R9.4.1 (ONT)	1000–2000 bp SE
Koeppel et al., 2022 [177]	eDNA sequencing	*Microcystis* spp. (non-target)	DNeasy PowerWater kit #	Long-read: eDNA	MinION (ONT)	N/A
Latz et al., 2022 [66]	18S rRNA gene V4, V6–V8 regions, 18S-ITS-28S rRNA gene	Algal groups: Stramenopiles, Alveolata, Archaeplastida, Hacrobia	ZymoBIOMICS DNA Miniprep kit (Zymo Research Corp) #, NucleoSpin Soil Kit (Macherey u nd Nagel) #	Short-read: 18S rRNA gene V4, V6–V8 regions * Long-read: 18S-ITS1-5.8S-ITS2-28S rRNA gene, V4_Balzano_F/V4F with 21R, 2742R and 3143R primers	Illumina MiSeq, Illumina NovaSeq 6000, PacBio Sequel	MiSeq: 2 × 300 bp PE NovaSeq: 2 × 150 bp PE PacBio: 4283 bp SE
O’Neill et al., 2022 [178]	18S full-length rRNA gene	341 genera, 982 species	High Pure PCR Template Preparation kit (Roche) #	Long-read: 18S full-length rRNA gene *	MinION (ONT)	MinION: ~1800 bp SE
He et al., 2023 [67]	18S rRNA gene V4 region, 18S full-length rRNA gene	118 species (37 species HAB-forming)	MicroElute Genomic DNA Kit (Omega) #	Short-read: 18S rRNA gene V4 region, 528F/706R primers Long-read: 18S full-length rRNA gene, 28F/42R primers	Illumina MiSeq, PacBio	MiSeq: 398 bp PacBio: 1789 bp SE
Hu et al., 2023 [179]	18S full-length rRNA gene	Algal genera: *Gonium*, *Pandorina*, *Volvulina*, *Platydorina*, *Colemanosphaera*, *Yamagishiella*, *Eudorina*, *Pleodorina*	DNA extraction Kit (Tiangen) #	Long-read: 18S full-length rRNA gene, Euk-A/Euk-B primers	PacBio Sequel II	1690–1825 bp SE
Gaonkar & Campbell, 2024 [68]	18S rRNA gene V4, V8–V9 regions, 18S full-length rRNA gene	Algal groups: Dinoflagellates, Diatoms, Chlorophyta, Rhodophyta, Haptophyta, Cryptophytes	AllPrep DNA/RNA MiniKit (Qiagen, USA)	Long-read: 18S full-length rRNA gene, SSUF/ITS-1dr primers Short-read: 18S rRNA gene V4, V8–V9 regions, Reuk454FWD1/ReukREV3 primers (V4), V8f/1510r primers (V8–V9)	MinION Mk1C (ONT), Illumina MiSeq	MinION: 1829 bp SE MiSeq: 2x300 bp PE
Judd et al., 2024 [29]	N/A	Algal microbiome: *Amphidinium carterae*	CTAB protocol	N/A	MinION, GridION, or PromethION (ONT)	N/A
Liu et al., 2024 [180]	18S full-length rRNA gene	Algal classes: Ulvophyceae, Chlorophyceae, Trebouxiophyceae	HP PlantDNA Kit (Omega Bio-Tek, Norcross, GR, USA)	Long-read: 18S full-length rRNA gene, Euk18SA/Euk18SB primers	PacBio Sequel	N/A
Meirkhanova et al., 2024 [181]	16S full-length rRNA gene	Synthetic algal-bacterial community: Cyanobacteria, Chlorophyta, Cryptophyta groups	DNEasy Power Water Kit (Qiagen, Hilden, Germany)	Long-read: 16S full-length rRNA gene, 27F/1492R primers	MinION Mk1C (ONT)	N/A
Mordret et al., 2024 [182]	18S-ITS-28S rRNA gene	73 phytoplankton strains	MasterPure Complete DNA and RNA Purification Kit (Epicenter Biotechnologies, USA), DNeasy Plant Pro Kit (Qiagen, USA)	Long-read: 18S-ITS1-5.8S-ITS2-28S rRNA gene, SSU-F and 3NDF with 21R, D3Ca-R primers	MinION R9.4.1 (ONT)	4391 bp SE
Baharudin et al., 2025 [26]	18S rRNA gene V7-V9 region, 18S-ITS-28S rRNA gene	Harmful dinophytes: *Pfiesteria piscicida*, *P. shumwayae*, *Luciella masanensis*, *Gyrodinium jinhaense*, *Malayana penaeicida* gen. *et* sp. nov.	Toyobo MagExtractor Plant Genome kit (Toyobo, Tokyo, Japan)	Short-read: 18S rRNA gene V7–V9 region, 18S-V7F/18S-V9R primers Long-read: 18S-ITS1-5.8S-ITS2-28S rRNA gene, 18ScomF1/D2C primers	Illumina MiSeq 2000, MinION R10.4.1 (ONT)	MiSeq: 2 × 300 bp PE MinION: >3000 bp SE
Gong et al., 2025 [21]	16S full-length rRNA gene	*Anabaena* spp., *Aphanizomenon* spp., *Cylindrospermum* spp., *Dolichospermum* spp., *Microcoleus*/*Phormidium* spp., *Lyngbya*/*Microseira* spp., *Microcystis* spp., *Nostoc* spp., *Synechocystis* spp., *Planktothrix* spp., *Pseudanabaena* spp.	DNeasy Blood & Tissue Kits (Qiagen, German-town, MD, USA), DNeasy PowerSoil Pro Kit (Qiagen) #	Long-read: 16S full-length rRNA gene, 27F/1492R primers	GridION R10 (ONT)	800–2000 bp SE
Marter et al., 2025 [183]	16S-ITS rRNA gene	Algal microbiome: *Coleofasciculus chthonoplastes*	DNeasy Blood and Tissue Kit (Qiagen, Hilden, Germany)	Long-read: 16S-ITS rRNA gene, 16S_27f/23S_130r primers	PacBio Sequel IIe	1827–3044 bp SE
Meirkhanova et al., 2025 [30]	16S full-length rRNA gene	Algal microbiomes: *Microcystis* spp., *Cryptomonas* spp.	DNEasy Power Water Kit (Qiagen, Hilden, Germany)	Long-read: 16S full-length rRNA gene, 27F/1492R primers	MinION Mk1C (ONT)	N/A
Mordret et al., 2025 [22]	18S-ITS-28S rRNA gene	*Prorocentrum nux*	DNeasy Plant Pro Kit (Qiagen, USA)	Long-read: 18S-ITS1-5.8S-ITS2-28S rRNA gene, SSU-F and 3NDF with 21R, D3Ca-R primers	MinION R9.4.1 (ONT)	N/A
Punnarak et al., 2025 [70]	18S rRNA gene V4 region and *coi* gene, 18S full-length rRNA gene	Algal classes: Bacillariophyceae, Dictyochophyceae, Dinophyceae, Haptophyceae, Pelagophyceae, Raphidophyceae	ZymoBIOMICS DNA Miniptep Kit (Zymo Research, CA, USA), E.Z.N.A. Soil DNA kit (Omega Bio-Tek, Norcross, GA, USA)	Short-read: 18S rRNA gene V4 region, TAReuk454FWD1/TAReukREV3 primers *coi* gene, mlCOIinfF/HCO2198 primers Long-read: 18S full-length rRNA gene, Euk-F/Euk-R primers	Illumina NovaSeq 6000, PacBio Sequel II	NovaSeq: 2 × 250 PE PacBio: ~1800 bp SE
Rousseau et al., 2025 [69]	16S rRNA gene V3–V4, V4–V5 regions, 16S full-length rRNA gene, 18S rRNA gene ITS1-ITS2 region	Algal microbiome: *Ascophyllum nodosum*	CTAB protocol	Short-read: 16S rRNA gene V3–V4, V4–V5 regions, 18S rRNA gene ITS2 regions, S-B-bact-0341-b-S-17F/799F_rc primers (V3-V4), 515F/926R primers (V4-V5), ITS1/PCR1-ITS4 primers (ITS2), 5,8S-FUN/ITS4-FUN primers (ITS2) Long-read: 16S full-length rRNA gene, 18S rRNA gene ITS1-ITS2 region, 27F/1492R primers (16S), ITS9-mum/LR3-I (ITS1-ITS2)	Illumina MiSeq, Illumina NovaSeq 6000, MinION Mk1C (ONT)	MiSeq: 2 × 300 bp PE NovaSeq: 2 × 250 bp PE MinION: 1326 bp SE, 1413 bp SE
Yu et al., 2025 [184]	16S full-length rRNA gene	Algal microbiome: *Microcystis aeruginosa*	CTAB protocol	Long-read: 16S full-length rRNA gene, N/A	PacBio	N/A

* where available the primer names were written, the full primer sequences can be accessed in the article; # manufacturer city/country information is unavailable for the given DNA extraction kit.
