# Supplementary material for: Systematic Review: Long-Read Sequencing in Algal Studies
Source: Int J Mol Sci. 2026 Mar 5;27(5):2415. doi: 10.3390/ijms27052415 (PMC12985685; doi:10.3390/ijms27052415)
Supplement: Supplementary file 1 [file ijms-27-02415-s001.zip › Table S1 Algal genomics.pdf]

Table S1. Summary of long-read algal genomics and associated assembly metrics.

Reference	Reported algae	Algal group	DNA extraction kit	Sequencing platforms	Assembly metrics (size, contigs, N50, BUSCOs (C))
Guo et al., 2019	*Nannochloropsis oceanica*	Ochrophyta	CTAB protocol	Sequel (PacBio),<br>HiSeq X (Illumina),<br>HiSeq 4000 (Illumina)	Size: 29.3 Mb<br>Contigs: 129<br>N50: 664.749 kb<br>BUSCO: 87.1 % (C)
Liu et al., 2020	*Mychonastes homosphaera*	Chlorophyta	Genomic DNA Extraction Kit (Qiagen)	Sequel (PacBio)	Size: 24.23 Mb<br>Contigs: 31<br>N50: 2 Mb<br>BUSCO: 89.4% (C)
Wang et al., 2020	*Pyropia yezoensis*	Rhodophyta	HMW DNA extraction from plant	RSII (PacBio),<br>HiSeq 2000 (Illumina),<br>Irys (Bionano)	Size: 108 Mb<br>Contigs: 660<br>N50: 340.8 kb<br>BUSCO: 84%
Zhang et al., 2020	*Chlamydomonas* sp. ICE-L	Chlorophyta	DNeasy Plant Mini Kit (Qiagen)	HiSeq X (Illumina),<br>GemCode (10X Genomics),<br>Sequel (PacBio)	Size: 541.86 Mb<br>Superscaffold (>100 bp): 946<br>N50: 19.23 Mb<br>BUSCO: 83.7% (C)
Dreher et al., 2021	Nostocales (ADA clade; 16 genomes)	Cyanobacteria	DNeasy PowerBiofilm extraction kit (Qiagen)	Sequel (PacBio),<br>HiSeq 3000 (Illumina)	16 genomes (9 C)<br>Size: 4.36-6.07 Mbp<br>Contigs:1 (C); Others: 4-286<br>N50: N/A<br>BUSCO: 94.19-99.89% (C)

Hulatt et al., 2021	*Diacronema lutheri* (former *Pavlova lutheri*)	Haptophyta	N/A	MiSeq (Illumina), Sequel (PacBio)	Size: 43.5 Mb<br>Contigs: 103<br>N50: 852.26 kbp<br>BUSCO: 80.80 % (C)
Chen et al., 2022	*Neoporphyra haitanensis*	Rhodophyta	CTAB protocol	Sequel (PacBio), HiSeq (Illumina)	Size: 49.67 Mb<br>Contig N50: 650 kb<br>Scaffold N50: 7.796Mb<br>BUSCO: 85.8%
Cho et al., 2023	6 genomes *Cyanidiophyceae*	Rhodophyta	CTAB protocol	Sequel II (PacBio), PromethION (ONT), HiSeq2500 (Illumina), NovaSeq6000 (Illumina)	6 genomes *Cyanidiophyceae*<br>Size: 8.79-16.5 Mb<br>Scaffolds: 20; 76-433<br>N50: 172.3-202.1 kbp<br>BUSCO: 94.1-96.7 % (C)
Gueidan et al., 2023	*Diplosphaera chodatii* strain CS-1475	Chlorophyta	Illumina: Phenol-chloroform and SDS/BME lysis buffer<br>PacBio: CTAB protocol	NextSeq 500 (Illumina), Sequel (PacBio)	Size: 85.6 Mb<br>Contigs: 62<br>N50: 2.626 Mb<br>BUSCO: 88.4% (C)
Chen et al., 2024	*Phaeocystis globosa*	Haptophyta	Plant DNA Mini Kit (Omega)	Sequel (PacBio), Illumina platform	Size: 129.7 Mb<br>Contigs: 396<br>N50: 569.7 kbp<br>Scaffolds: 85; N50 scaff.: 6601.8 kbp<br>BUSCO: 80.9 % (C)
Jian et al., 2024	*Prymnesium parvum* strains CCMP 3037 and UTEX 2797	Haptophyta	DNeasy Plant Kit (Qiagen)	MGISEQ-2000 (MGI), Sequel II (PacBio)	Size: UTEX 2797 - 97.56 Mb<br>CCMP 3037 - 107.32 Mb<br>Contigs: 463 & 362<br>N50: 596.99 kbp & 968.39 kbp

					Scaffolds: 34; N50 scaff.: 5.35 Mb & 3.61 Mb<br>BUSCO: 81.2% (C) & 77.2% (C)
Moretto et al., 2024	*Umezakia ovalisporum* BLCC-F208, *Microcystis sp.* BLCC-F209, *Microcystis sp.* BLCC-F210, *Pelatocladus sp.* BLCC-F211, *U. ovalisporum* BLCC-F215, *Raphidiopsis* BLCC-F218	Cyanobacteria	DNeasy PowerSoil Kit (Qiagen)	HiSeq (Illumina), N/A (ONT)	Size: 4.9 Mb<br>Contigs: 1(ONT); 55-349 (Illumina)<br>N50: 4.9 Mb (ONT); 22.4-135.4 kbp (Illumina)<br>BUSCO (ONT): 99.1% (C),<br>BUSCO (Illumina): 85 -99.96 % (C)
Raymond et al., 2024	*Sanguina aurantia*<br>*C.reinhardtii* (reference)	Chlorophyta	Plant DNA Isolation Kit (Norgen Biotek)	PromethION (ONT), HiSeq (Illumina)	A & B genomes<br>Size: 96 Mb & 102 Mb<br>Scaffolds: 38 & 50<br>N50: 5.42 Mb & 6.41 Mb<br>BUSCO: 87.4% (C) & 81.9% (C):98.9% (ref.)
Petroll et al., 2025	*Bostrychia moritziana*	Rhodophyta	CTAB protocol	MinION (ONT),<br>Sequel II (PacBio),<br>NextSeq 2000 (Illumina)	Size: 1.04 Gb<br>Contigs: 32<br>N50: N/A<br>BUSCO: 74.5% (C)

Abbreviations: CTAB - cetyltrimethylammonium bromide; HMW - high molecular weight; (C) - genome completeness %; N/A - not available.

References:

Chen, H.; Chu, J.S.-C.; Chen, J.; Luo, Q.; Wang, H.; Lu, R.; Zhu, Z.; Yuan, G.; Yi, X.; Mao, Y.; et al. Insights into the Ancient Adaptation to Intertidal Environments by Red Algae Based on a Genomic and Multiomics Investigation of Neoporphyra Haitanensis. *Mol Biol Evol* **2022**, *39*, msab315, doi:10.1093/molbev/msab315.

Chen, N.; Xu, Q.; Zhu, J.; Song, H.; He, L.; Liu, S.; Song, X.; Yuan, Y.; Chen, Y.; Cao, X.; et al. Chromosome-Scale Genome Assembly Reveals Insights into the Evolution and Ecology of the Harmful Algal Bloom Species Phaeocystis Globosa Scherffel. *iScience* **2024**, *27*, doi:10.1016/j.isci.2024.110575.

Cho, C.H.; Park, S.I.; Huang, T.-Y.; Lee, Y.; Ciniglia, C.; Yadavalli, H.C.; Yang, S.W.; Bhattacharya, D.; Yoon, H.S. Genome-Wide Signatures of Adaptation to Extreme Environments in Red Algae. *Nat Commun* **2023**, *14*, 10, doi:10.1038/s41467-022-35566-x.

Dreher, T.W.; Davis, E.W.; Mueller, R.S. Complete Genomes Derived by Directly Sequencing Freshwater Bloom Populations Emphasize the Significance of the Genus Level ADA Clade within the Nostocales. *Harmful Algae* **2021**, *103*, 102005, doi:10.1016/j.hal.2021.102005.

Gueidan, C.; Mead, O.L.; Nazem-Bokaee, H.; Mathews, S. First Draft of an Annotated Genome for a Lichenised Strain of the Green Alga Diplosphaera Chodatii (Prasiolales, Trebouxiophyceae). *European Journal of Phycology* **2023**, *58*, 427–437, doi:10.1080/09670262.2023.2165711.

Guo, L.; Liang, S.; Zhang, Z.; Liu, H.; Wang, S.; Pan, K.; Xu, J.; Ren, X.; Pei, S.; Yang, G. Genome Assembly of Nannochloropsis Oceanica Provides Evidence of Host Nucleus Overthrow by the Symbiont Nucleus during Speciation. *Commun Biol* **2019**, *2*, 249, doi:10.1038/s42003-019-0500-9.

Hulatt, C.J.; Wijffels, R.H.; Posewitz, M.C. The Genome of the Haptophyte Diacronema Lutheri (Pavlova Lutheri, Pavlovales): A Model for Lipid Biosynthesis in Eukaryotic Algae. *Genome Biol Evol* **2021**, *13*, evab178, doi:10.1093/gbe/evab178.

Jian, J.; Wu, Z.; Silva-Núñez, A.; Li, X.; Zheng, X.; Luo, B.; Liu, Y.; Fang, X.; Workman, C.T.; Larsen, T.O.; et al. Long-Read Genome Sequencing Provides Novel Insights into the Harmful Algal Bloom Species *Prymnesium Parvum*. *Science of The Total Environment* **2024**, *908*, 168042, doi:10.1016/j.scitotenv.2023.168042.

Liu, C.; Shi, X.; Wu, F.; Ren, M.; Gao, G.; Wu, Q. Genome Analyses Provide Insights into the Evolution and Adaptation of the Eukaryotic Picophytoplankton Mychonastes Homosphaera. *BMC Genomics* **2020**, *21*, 477, doi:10.1186/s12864-020-06891-6.

Moretto, J.A.; Berthold, D.E.; Lefler, F.W.; Mazzei, V.; Loftin, K.A.; Laughinghouse IV, H.D. Genome Sequences of Toxigenic Cyanobacteria from a Bloom in Lake Mattamuskeet, North Carolina (United States). *Journal of Phycology* **2024**, *60*, 1349–1355, doi:10.1111/jpy.13523.

Petroll, R.; West, J.A.; Ogden, M.; McGinley, O.; Craig, R.J.; Coelho, S.M.; Borg, M. The Expanded Bostrychia Moritziana Genome Unveils Evolution in the Most Diverse and Complex Order of Red Algae. *Current Biology* **2025**, *35*, 2771-2788.e8, doi:10.1016/j.cub.2025.04.044.

Raymond, B.B.; Guenzi-Tiberi, P.; Maréchal, E.; Quarmby, L.M. Snow Alga Sanguina Aurantia as Revealed through de Novo Genome Assembly and Annotation. *G3 Genes|Genomes|Genetics* **2024**, *14*, jkae181, doi:10.1093/g3journal/jkae181.

Wang, D.; Yu, X.; Xu, K.; Bi, G.; Cao, M.; Zelzion, E.; Fu, C.; Sun, P.; Liu, Y.; Kong, F.; et al. Pyropia Yezoensis Genome Reveals Diverse Mechanisms of Carbon Acquisition in the Intertidal Environment. *Nat Commun* **2020**, *11*, 4028, doi:10.1038/s41467-020-17689-1.

Zhang, Z.; Qu, C.; Zhang, K.; He, Y.; Zhao, X.; Yang, L.; Zheng, Z.; Ma, X.; Wang, X.; Wang, W.; et al. Adaptation to Extreme Antarctic Environments Revealed by the Genome of a Sea Ice Green Alga. *Current Biology* **2020**, *30*, 3330-3341.e7, doi:10.1016/j.cub.2020.06.029.
