# Supplementary material for: Systematic Review: Long-Read Sequencing in Algal Studies
Source: Int J Mol Sci. 2026 Mar 5;27(5):2415. doi: 10.3390/ijms27052415 (PMC12985685; doi:10.3390/ijms27052415)
Supplement: Supplementary file 1 [file ijms-27-02415-s001.zip › Table S2 Algal Pangenome Studies.pdf]

Table S2. Summary of algal pangenome studies.

Reference	Study system (# genomes)	Algal group	Geographical sources	Sequencing platforms	Species delimitation criteria (ANI, dDDH)	Pangenome construction structure
Rossoni et al., 2019	*Galdieria sulphuraria* (9), *Cyanidioschyzon merolae* (2), *Galdieria phlegrea* (2)	Rhodophyta	Europe (7), USA (4), Asia (2)	RSII (PacBio)	N/A	Pangenome: 9075 orthogroups
Cao et al., 2022	*Microcystis* (23)	Cyanobacteria	Japan(9), China(6), South Korea(1), USA(2), Netherlands(1), Unknown(4)	N/A	ANI > 0.95	Open pangenome: 22,009 genes<br>Core: 7.5%<br>Accessory: 39.4%<br>Unique: 53.1%
Gann et al., 2022	*Aureococcus anophagefferens* (5)	Ochrophyta	USA (5)	NextSeq500 (Illumina), MinION Mk1B (ONT)	N/A	Pangenome: 4278 functional orthologs<br>Core: 73.68%<br>Accessory/unique: 26.32%
Cai et al., 2023	*Microcystis* (122)	Cyanobacteria	USA & Canada(69), East Asia(35), Brazil(8), Europe(6), Africa(3), Oceania(1)	N/A	within-cluster (ANI >= 0.970, dDDH > 0.750)<br>between-cluster (ANI <0.970, dDDH<0.750)	Open pangenome: 21880 genes<br>Core: 7.5%<br>Accessory: 66.6%<br>Unique: 25.9%
Wisecaver et al., 2023	*Prymnesium parvum* strains UTEX2797 and 12B1(USA), and 13 strains	Haptophyta	USA; different geographical regions	Novaseq 6000 (Illumina), HiSeq	N/A	Pangenome: 47,043 orthogroups<br>Core: 34.9%

				(Illumina),<br>MinION R9 (ONT)		Accessory: 44.2%<br>Unique: 20.9%
Sibbald et al., 2025	*Aureococcus anophagefferens* (5),<br>*Aureoumbra lagunensis* (1),<br>*Pelagomonas calceolata* (1)	Ochrophyta	Canada	Novaseq 6000 (Illumina), HiSeq (Illumina),<br>MinION (ONT)	ANI > 0.97	Pangenome: 23,356 orthogroups<br>Core: 81.1%<br>Accessory/unique: 18.9%

Abbreviations: N/A - not available for this study; ANI - average nucleotide identity; dDDH - digital DNA-DNA hybridization.

References:

Cai, H.; McLimans, C.J.; Beyer, J.E.; Krumholz, L.R.; Hambright, K.D. Microcystis Pangenome Reveals Cryptic Diversity within and across Morphospecies. *Science Advances* **2023**, *9*, eadd3783, doi:10.1126/sciadv.add3783.

Cao, H.; Xu, D.; Zhang, T.; Ren, Q.; Xiang, L.; Ning, C.; Zhang, Y.; Gao, R. Comprehensive and Functional Analyses Reveal the Genomic Diversity and Potential Toxicity of Microcystis. *Harmful Algae* **2022**, *113*, 102186, doi:10.1016/j.hal.2022.102186.

Gann, E.R.; Truchon, A.R.; Papoulis, S.E.; Dyhrman, S.T.; Gobler, C.J.; Wilhelm, S.W. Aureococcus Anophagefferens (Pelagophyceae) Genomes Improve Evaluation of Nutrient Acquisition Strategies Involved in Brown Tide Dynamics. *Journal of Phycology* **2022**, *58*, 146–160, doi:10.1111/jpy.13221.

Rossoni, A.W.; Price, D.C.; Seger, M.; Lyska, D.; Lammers, P.; Bhattacharya, D.; Weber, A.P. The Genomes of Polyextremophilic Cyanidiales Contain 1% Horizontally Transferred Genes with Diverse Adaptive Functions. *eLife* **2019**, *8*, e45017, doi:10.7554/eLife.45017.

Sibbald, S.J.; Lawton, M.; Maclean, C.; Roger, A.J.; Archibald, J.M. Pangenome Biology and Evolution in Harmful Algal-Bloom-Forming Pelagophyte Algae. *Current Biology* **2025**, *35*, 4215. DOI: 10.1016/j.cub.2025.07.055.

Wisecaver, J.H.; Auber, R.P.; Pendleton, A.L.; Watervoort, N.F.; Fallon, T.R.; Riedling, O.L.; Manning, S.R.; Moore, B.S.; Driscoll, W.W. Extreme Genome Diversity and Cryptic Speciation in a Harmful Algal-Bloom-Forming Eukaryote. *Current Biology* **2023**, *33*, 2246-2259.e8, doi:10.1016/j.cub.2023.05.003.
